# Prevalence of meconium-stained amniotic fluid and factors associated with emergency cesarean section: multicenter cross-sectional study in south central Ethiopia

**DOI:** 10.3389/fgwh.2024.1516665

**Published:** 2024-12-23

**Authors:** Temesgen Tantu, Biruk Melkamu, Muluken Gunta, Tayech Tantu, Yohanna Aregawi, Dereje Gashaw

**Affiliations:** ^1^ College of Medicine and Health Sciences, Wolkite University, Wolkite, Ethiopia; ^2^ Department of Obstetrics and Gynecology, University of Rwanda, Kigali, Rwanda; ^3^ College of Medicine and Health Sciences, Wachamo University, Hosanna, Ethiopia; ^4^ Wolaita Zone Health Department, Sodo, Ethiopia; ^5^ Department of Pediatrics, Betezaida Hospital, Aleta Wendo, Sidama Regional State, Ethiopia; ^6^ Department of Surgery, College of Medicine and Health Sciences, Saint Paul's Hospital Millennium Medical College (SPHMMC), Addis Ababa, Ethiopia; ^7^ Department of Surgery, University of Rwanda, Kigali, Rwanda; ^8^ College of Medicine and Health Sciences, Bahirdar University, Bahirdar, Ethiopia; ^9^ Department of Surgery, University of Rwanda, Kigali, Rwanda

**Keywords:** MSAF, fetal distress, MAS, obstructed labor, perinatal asphyxia, eclampsia

## Abstract

**Background:**

Meconium is thick black-green fetal intestinal content starting from the early first trimester of gestation. Unfortunately, if it is released into the amniotic cavity due to any cause, it can be associated with neonatal mortality and morbidity.

**Objective:**

To identify the factors associated with meconium-stained amniotic fluid among mothers undergoing emergency cesarean section in specialized hospitals cross-sectional study in south central Ethiopia from August 1, 2022, to 30, October 2022

**Method:**

Institution based multicenter cross-sectional study was done prospectively through meticulous chart review and interview on 875 mothers who gave birth with emergency cesarean section. Data were entered using Epi data 7 and analyzed with SPSS 26. The association between independent variables and meconium-stained amniotic fluid was estimated using an odds ratio with 95% confidence intervals. The statistical significance of the association was declared at *P*-value < 0.05

**Result:**

The prevalence of meconium-stained amniotic fluid is 31.4%. Gravidity (AOR 3.643; 95% CI:1.215,10.921), time interval between decision to delivery (AOR 1.361; 95% CI: 0.424,4.365),eclampsia(AOR 8.022; 95% CI: 1.634,39.372), time taken from referring institution to managing institution (AOR 15.258; 95% CI: 1.591,146.328), obstructed labor (AOR 24.614; 95% CI: 6.073,99.766), cephalopelvic disproportion (AOR 2.640; 95% CI:1.002,6.950), fetal heart beat abnormality (fetal bradycardia AOR 2.068; 95%CI 0.997,4.292) (fetal tachycardia AOR 3.513; 95% CI:1.633,7.556) duration of labor(AOR 5.44; 95% CI: 1.355,9.782) and referral from health center(AOR 5.41; 95% CI: 2.053,14.272) are associated with MSAF whereas obstetric complications(AOR 6.820; 95% CI: 2.870,16.202), cesarean section scar (AOR 3.010; 95% CI: 1.344,6.740) are negatively associated.

**Conclusion:**

Prevalence of meconium-stained amniotic fluid is relatively high for which intrapartum, obstetric and institution related factors are incriminated. Therefore, an improvement in the quality of antenatal and intrapartum care is strongly recommended; professional development at the health center, building the infrastructure, and strengthening the referral system is also suggested.

## 1 Background

Amniotic fluid is the clear liquid in the uterine cavity that encircles the fetus from the first weeks until the late weeks of gestation. Throughout pregnancy, the fetus is almost the sole source of it. It has multiple physiological, biochemical, and physical functions, which have critical roles in growth and development *in utero* (1). Meconium is a thick, sterile, greenish material found in the intestine of a growing fetus around the last trimester of pregnancy. The color of meconium is secondary to the bile content, and meconium comprises intestinal desquamated cells, mucous, fatty materials, lanugo, blood group-specific glycoproteins, intestinal secretion, gastrointestinal mucin, and fatty material from the vernix caseosa (2, 3).

Fetal defecation into the amniotic fluid cavity usually starts from the early first trimester of gestation and progressively becomes infrequent after 20 weeks, with parallel maturation of anal sphincter innervation; otherwise, commonly, fetal defecation happens 12 h–24 h after delivery (4, 5). However, a stressful condition *in utero* can induce the passage of meconium due to increased bowel movement and anal sphincter relaxation secondary to vagal stimulation associated with cord compression or sympathetic impulse during the hypoxic state.

Additionally, 20%–30% of fetuses with meconium-stained amniotic fluid (MSAF) have neurologic and respiratory depression at birth, which indirectly indicates chronic insult (6–9). The incidence of MSAF can vary based on gestational age, and according to multiple studies, it ranges from 5.1% in preterm fetuses to 27.1% in post-term fetuses, which has similar reports from low-resource countries; however, the prevalence MSAF among mothers who underwent emergency cesarean section has not been studied (10–18). Although meconium is sterile, the mucopolysaccharide content is fertile ground for the growth of bacteria, especially Escherichia coli (19).

It has an inhibitory effect on the phagocytosis of polymorphonuclear cells. Moreover, the meconium content can induce pronounced inflammatory reactions in fetal and neonatal lungs, which causes chemical pneumonitis, alveolar collapse, and cellular apoptosis (19, 20). Furthermore, depending on the thickness of the meconium, it can obstruct the upper airway tract after delivery during the first breath of life. According to studies, the rate of perinatal asphyxia was 20%–33% (21), and meconium aspiration syndrome (MAS) was 2%–10% in infants born with MSAF (21). Of the cases with MSAF, there were deaths (4.22%), mechanical ventilation (29.7%) requirements, and pneumothorax (11.53%) (9, 13, 16). Therefore, it has a significant effect on neonatal mortality and morbidity (14–18, 22).

Ethiopia is one of the low-income countries with the highest burden of maternal, neonatal, and infant mortality and morbidity in the world. Recent evidence shows that Ethiopia still faces a considerable burden of newborn morbidity and mortality despite the substantial improvements in access to and availability of obstetric caregiving facilities (18, 23). The neonatal mortality associated with meconium aspiration syndrome is 3%–5%. There are controversial mechanisms such as airway obstruction, alveolar or parenchymal inflammation, impaired surfactant production and function, infection, and direct toxicity of meconium constituents. It has significant adverse health complications like the need for mechanical ventilatory support (43.1%), respiratory and metabolic acidosis (30.6%), pulmonary hypertension (11.1%), and hypoxic-ischemic encephalopathy (29.2%) (18, 22, 24). The pooled prevalence of perinatal asphyxia is around 24% according to a systematic review, and MSAF is one of the strong risk factors responsible for this high prevalence (25). Perinatal asphyxia is one of the complications associated with MSAF, which has adverse health consequences like neonatal death, encephalopathy, respiratory, and other organ complications (26).

There are a variety of factors associated with the prevalence of MSAF, according to multiple studies across the scientific world, including different Ethiopian studies. Most of them are intrapartum-related, such as labor duration, fetal distress, obstructed labor, and the mechanism of the onset of labor; obstetric complications like preeclampsia, post-term, and others (6, 10–14, 18, 27–29). So, studying these factors in detail has a substantial impact on identifying and implementing strategies to improve obstetric care; thereby, the contribution of MSAF to neonatal morbidity and mortality will progressively be controlled.

Finally, there is a shortage of data on factors associated with MSAF in low-resource areas, especially after an emergency cesarean section. MSAF in the early phase of labor is becoming one of the commonest indications for emergency cesarean sections in contemporary obstetric practice because of its aforementioned adverse complications in neonatal health outcomes (13, 16). Compared to clear amniotic fluid, mothers with MSAF have a higher preponderance for emergency cesarean sections (30).

While there are numerous studies focusing on various routes of delivery, to the best of the authors’ knowledge, there has been no study specifically examining the prevalence of Meconium-Stained Amniotic Fluid (MSAF) following emergency cesarean sections. Therefore, the primary aim of this study was to assess the prevalence of MSAF among mothers undergoing emergency cesarean sections and the secondary aim was to identify the factors associated with MSAF in these cases. Furthermore, most studies do not include institutionally related delays as a factor, but those have a negative impact on obstetric and neonatal outcomes in low-income countries. Hence, this study included those delays as a factor, as there is also no study that has been done to identify factors after emergency cesarean delivery in the area.

## 2 Materials and methods

### 2.1 Study area, design, and populations

The multi-center cross-sectional study was conducted at two institutions in south central Ethiopia: Wachamo University Comprehensive Hospital and Wolkite University Comprehensive Hospital, August 1, 2022, to October 30, 2022. The southern nation has three comprehensive specialized hospitals, of which two are located in south central region; we selected those two. Wolkite University specialized hospital is located in Wolkite city, which is 160 km from the capital of Ethiopia, and it has a total of 200 deliveries in a month; the rate of cesarean sections is 30%. Furthermore, Wachamo University specialized hospital is located in Hossaina city, which is 200 km from the capital of Ethiopia, and it has a total of 500 deliveries per month with a cesarean section rate of 38%. All pregnant ladies who gave birth through emergency cesarean delivery at these hospitals during the study period and who were eligible were included.

### 2.2 Sample size determination and sampling technique

We applied the single proportion formula for the first objective and the double proportion formula for the second objective, selecting the variable with the larger sample size for the study. The single proportion formula, which resulted in a large sample size, was utilized to estimate the sample size based on previous studies conducted in Bahirdar, Ethiopia, where the prevalence of meconium-stained amniotic fluid was found to be 24% (10)., 95% CI, and 3% margin of Error;

By using a formula for calculating

$n=\frac{z\alpha^{2}p(1-p)}{d^{2}}$

Where *n* = sample size, *z* = confidence interval, *p* = estimate of proportion, d = margin of error. Then, a 10% non-response rate was included, and the final sample size becomes 875. The sample size was distributed proportionally among the hospitals based on their monthly deliveries through emergency cesarean sections. After calculating the rate of monthly cesarean sections at individual hospitals, the total sample distribution for the two hospitals was as follows: for Wachamo University Comprehensive Hospital, 621, and for Wolkite, 254. All emergency cesarean deliveries were taken until it reached the desired sample number.

### 2.3 Inclusion and exclusion criteria

#### 2.3.1 Inclusion criteria

All mothers who gave birth through emergency cesarean section for reasons such as fetal distress, obstructed labor, cephalopelvic disproportion, antepartum hemorrhage, malpresentation in labor, failed induction, scar dehiscence, cord prolapse, meconium-stained amniotic fluid, and labor abnormalities at each stage (such as arrest of cervical dilatation, prolonged latent phase of labor, prolonged second stage of labor, arrest of descent, and protracted cervical dilatation) are included.

#### 2.3.2 Exclusion criteria

Mothers who underwent emergency cesarean delivery due to one of the following conditions: a fetus diagnosed with a severe lethal congenital anomaly either before or after the procedure, absence of a fetal heartbeat upon admission, breech presentation, or mothers who were unable or chose not to provide their medical history due to any medical or obstetric complications.

Operational definitions:

Duty hours: it is a time period from 5:30 PM−8:00 AM.

Antepartum hemorrhage: Ante-partum hemorrhage (APH) is vaginal bleeding from the 28th week of gestation till the fetus (last fetus in case of multiple pregnancies) is delivered.

Failed induction: Failure to achieve regular (e.g., every 3 min) uterine contractions and cervical change after at least 6–8 h of the maintenance dose of oxytocin administration, with artificial rupture of membranes if feasible.

Cord prolapse: Umbilical cord (UC) descends alongside or beyond the fetal presenting part in the presence of ruptured membranes.

Scar dehiscence: Separation or disruption of the previous cesarean section scar.

Prolonged latent phase of labor: it is more than 20 h for primigravida's and more than 14 h in multiparas.

Protracted cervical dilatation: the rate of cervical dilatation less than 1 cm/h for the four hours.

Arrest of cervical dilation: No cervical change for 4 h in active phase of labor.

Prolonged second stage of labor: more than 3 h and 2 h for primigravida and multigravidas without the epidural anesthesia.

### 2.4 Data collection instruments

The investigators informed the participants about the purpose and usefulness of the study, and mothers who agreed to participate gave their informed written consent. After obtaining it, data collection was subsequently commenced. The principal investigator supervised the data collection process while trained midwives collected the data. The data collectors conducted the collection through detailed interviews and reviews of the file folders of individual patients. A well-structured data extraction tool was developed in English after reviewing the existing literature and was pretested at Butajira Hospital with a 5% sample size for subsequent modifications. The content of the extraction tool included sociodemographic, obstetric, intrapartum, institutional, indications for cesarean section, and intraoperative factors like the type of anesthesia, the operating surgeon, and skin incision to delivery. Moreover, semi-structured, detailed interviews were employed to further explore the sociodemographic and institution-related factors. Institution-related factors are factors like the time taken from decision to delivery, the time taken from the referring institution to the managing institution, the place of referral, and the type of admission (referred case or not).

### 2.5 Data processing and analysis

The collected data was checked, cleared, and entered into EP Info version 7 and then exported to SPSS version 26 for further clearance and analysis. By running frequency distribution, data were rechecked for any missing variables during entry. The results were presented through descriptive analysis, frequency distribution, graphs, tables, and means. All variables were analyzed with bivariate logistic regression independently to evaluate any association with the outcome variables. Afterward, those variables with *p* values less than 0.05% and 95% confidence intervals were analyzed with multivariate logistic regression to further identify the strength of association with the dependent variables. Finally, a significant association was obtained for the *p*-value < 0.05% and 95% CI. Model fitness was measured using the Hosmer and Lemeshow goodness of fit measures and the Nagelkerke R Square: 0.64 and 0.58, respectively. The variance inflation factor (VIF > 10) was used to test for multicollinearity between the explanatory variables.

### 2.5 Variables

Dependent variables: meconium-stained amniotic fluid.

Independent variables: Sociodemographic factors: maternal age, occupation, residency, income, educational status. Obstetric factors: parity, gravidity, antepartum complication, ANC(ante natal care) follow up, place of ANC follow up, previous obstetrics experience, previous history of abortion, fetal presentation, fetal number, Institution related factors: place of referral, place of ANC follow up, type of admission, decision to delivery Intraoperative and perioperative factors: surgeon, intraoperative maternal blood pressure, incision to delivery, Intrapartum factors: stage of labor, sex of neonate, duration of labor, time of operation, labor abnormalities, weight of neonate.

## 3 Results

### 3.1 Sociodemographic characteristics

In total, 875 pregnant women were selected during three months of collection. Most of the respondents (764, 87.3%) were in the age range of 20–34 years, with the mean age of the mothers being 26 (95% CI; 20, 32). Almost all respondents (856, 97.8%) were married, and nearly 80% of respondents attended a minimum of elementary school. (Table 1).

**Table 1:** Sociodemographic characteristics of mothers.

Variables		*N* = 875	%	*X* ^2^	*p*
Family income per month (Eth.birr)	<1,000	270	30.9	82	0.67
	1,001–4,999	364	41.6		
	>5,000	241	27.5		
Occupation (mother)	Farmer	149	17.0	20	0.9
	Merchant	112	12.8		
	Government employee	166	19.0		
	House wife	432	49.4		
	Others	16	1.8		
Age(years)	<20	47	5.4	12	0.8
	≥35	64	7.3		
	20–34	764	87.3		
Place of residence	Rural	295	33.7	4	0.4
	Urban	580	66.3		
Current marital status	Married	856	97.8	13	0.32
	Unmarried	16	1.8		
	Divorced	3	.3		
Level of education	Elementary level	263	37.4	22	0.6
	High school level	244	34.7		
	Diploma level	82	11.7		
	Degree level and above	114	16.2		

### 3.2 Health institution and obstetrics related characteristics

**Figure 1:**
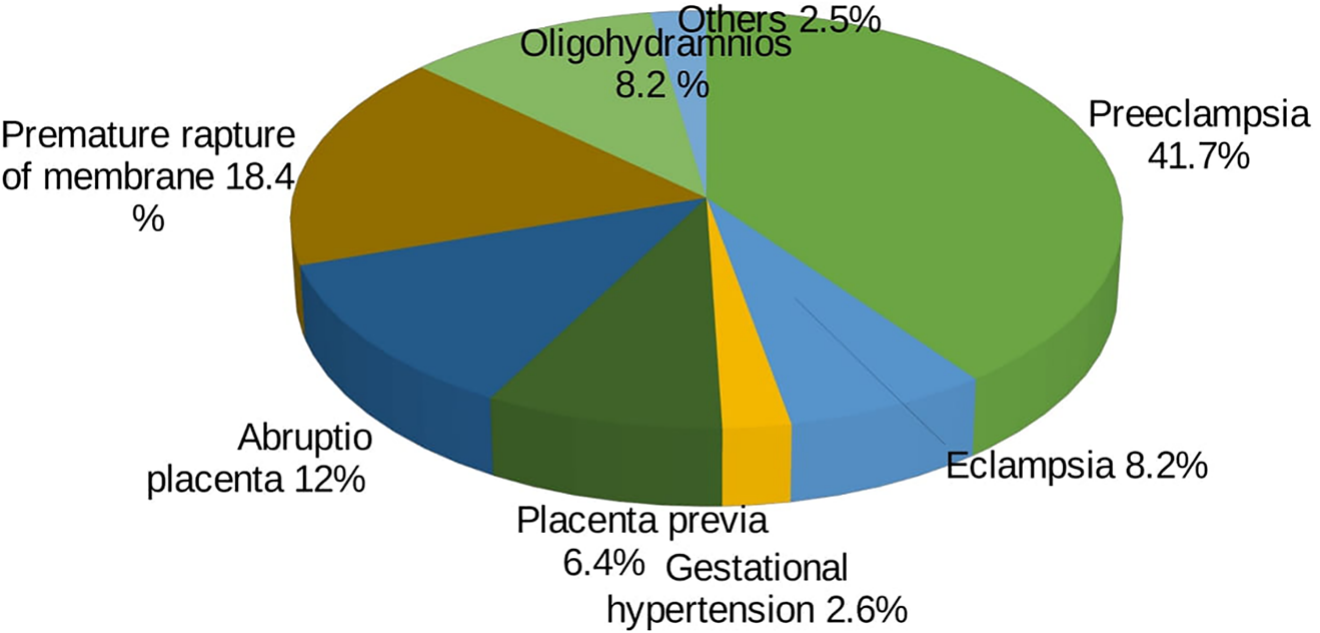
Frequency of preoperative obstetric compilations.

From all the respondents, more than 98% (863) have ANC follow-up, and 81.5% (703) of them have the ANC follow-up either at a private clinic or at a health center. Additionally, 1.5% (13) and 7.5% (66) of women have eclampsia and preeclampsia, respectively. Term pregnancy comprises 85.7% (750) of the pregnancies, and 26.1% of the respondents have had a previous cesarean section. Of all cases, 53% were referred from other health institutions, and 61.7% of referrals were from government hospitals (Table 2). Among the obstetric complications, preeclampsia (41.7%), premature rupture of membranes (18.4%), and eclampsia (8.2%) are the commonest (Figure 1).

**Table 2:** Health institution and obstetric related factors.

Variables		*N* (875)	%	*X* ^2^	*p*
Gestational age in weeks	Preterm (<37 weeks)	39	4.5	8	0.63
	term (37–42weeks)	750	85.7		
	post term (>42 weeks)	86	9.8		
Previous history of abortion	Yes	61	7.0	3	0.72
	No	814	93.0		
Previous history of still birth	Yes	22	2.5	4	0.51
	No	853	97.5		
Previous history of early neonatal death	Yes	13	1.5	2	0.29
	No	862	98.5		
ANC in the current pregnancy	Yes	863	98.6	11	0.27
	No	12	1.4		
Place of ANC	Health center or private clinic	703	81.5	9	0.31
	primary hospital	139	16.1		
	Referral Hospital	21	2.4		
History of previous uterine scar	No	647	73.9	10	0.04
	Yes	228	26.1		
Number of previous uterine scar	One	148	64.9	11.22	0.26
	Two	72	31.6		
	Three and above	8	3.5		
Type of pregnancy by fetal number	Singleton	875	100	8.45	
Preoperative maternal and/or fetal obstetric complications	Yes	158	18.1	2.99	0.01
	No	717	81.9		
Preoperative maternal medical complications	Yes	27	3.1	8	0.85
	No	848	96.9		
Admission type by referral	Not referred	464	53.0	4.56	0.37
	Referred	411	47.0		
Place of referral	Health center	84	18.1	5.99	0.05
	Government hospital	287	61.9		
	Private clinic or Hospital	93	20.0		
Onset of labor	Spontaneous	800	91.4	4.4	0.50
	Induced	75	8.6		
Eclampsia	Yes	13	1.5	12.33	0.002
	No	862	98.5		
Preeclampsia	Yes	66	7.5	11	0.86
	No	809	92.5		
Gravidity	Gravida 1	339	38.7	7.4	0.1
	Gravida 2–4	442	50.5		
	Gravida 5 and above	94	10.7		

### 3.3 Perioperative and intrapartum related characteristics

**Figure 2:**
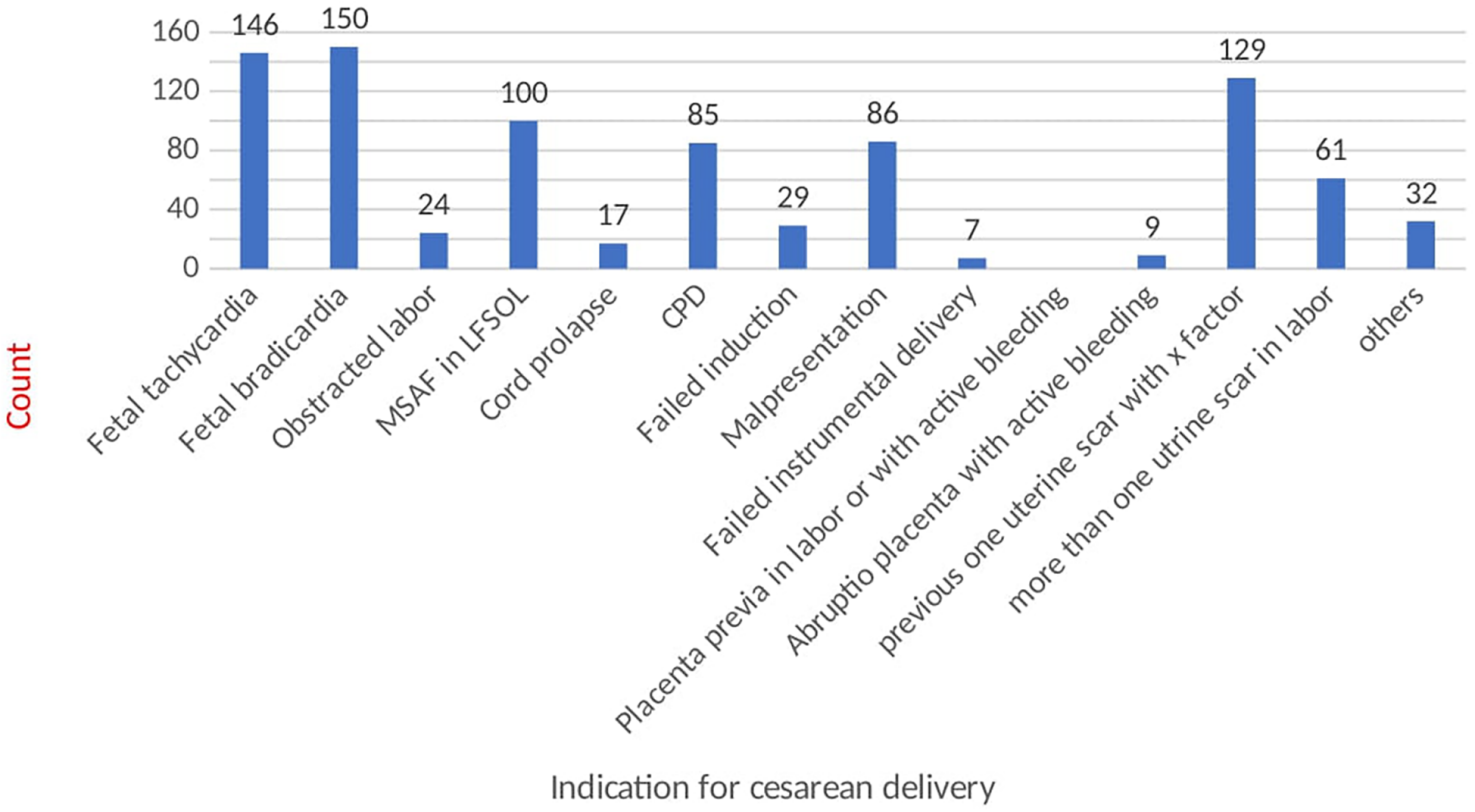
Indication of cesarean section.

The amniotic fluid is stained with meconium in 275 (31.4%) cases. Most of the cases have vertex (86.4%) presentation and are admitted in the latent phase of labor (50.5%). For the referred cases, the mean duration of time taken for transport was 2.0 (SD ± 1.77) hours. There are 39 (4.5%) cases with obstructed labor and 45 (5.1%) cases with cephalopelvic disproportion. Moreover, around 58% of operations were done during duty hours; of these, more than 90% of surgeries were performed by the second-year and third-year resident physicians (Table 3). The commonest indications for emergency cesarean section are as follows: non-reassuring fetal heart rate (34%), malpresentations (10.4%), and previous cesarean scar (14%) (Figure 2).

**Table 3:** Intrapartum and preoperative characteristics.

Variables		Frequency	Valid percent	*X* ^2^	*P*
Fetal presentation	Vertex	756	86.4	3.2	0.79
	Brow	9	1.0		
	Face	23	2.6		
	Breech	71	8.1		
	Shoulder	16	1.8		
Time of operation	Working hours	360	41.1	16.49	0.12
	Duty hours	515	58.9		
Type of anesthesia	Spinal anesthesia	808	92.3	1.3	0.22
	General anesthesia	67	7.7		
Maternal blood pressure at decision for operation (SBP/DBP)	100–139/60–89	686	78.4	2.1	0.17
	140/90 and above	189	21.6		
Maternal blood pressure after anesthesia, but before fetal extraction	<100/60	27	3.1	4.5	0.18
	100–139/60–89	743	84.9		
	140/90 and above	105	12.0		
Type of skin incision	Pfannenstiel	826	94.4	4.2	0.8
	Midline	49	5.6		
Surgeon	Year one resident	21	2.4	3.8	0.39
	Year two resident	514	58.7		
	Year three resident	322	36.8		
	Year four resident	13	1.5		
	Senior	5	0.6		
Sex of neonate	Male	484	55.3	6.7	0.31
	Female	391	44.7		
Neonatal weight range	1,000–1,499 g	4	0.5	2.1	0.21
	1,500–2,499 g	75	8.6		
	2,500–3,999 g	704	80.5		
	4,000 g and above	92	10.5		
Interval b/n decision to delivery	Greater than 60 min	380	43.4	8.7	0.02
	30–60 min	417	47.7		
	Less than 30 min	78	8.9		
Stage of labor	Second stage of labor	99	38.7	13.8	0.04
	Active phase labor	286	50.5		
	Latent phase of labor	490	10.7		
Duration Labor	Greater than or equal 24 h.	67	7.7	8.9	0.022
	Less than 24 h	808	92.3		
Time taken from referring to managing institution	Less than 1 h.	424	48.5	5.1	0.03
	More than 1 h.	450	51.5		
Cephalopelvic disproportion	Yes	45	5.1	7.4	0.001
	No	830	94.9		
Obstructed labor	Yes	39	4.5	8.8	0.02
	No	836	95.5		
Meconium-stained amniotic fluid	Clear	600	68.6		
	Meconium stained	275	31.4		

### 3.4 Factors associated with meconium-stained amniotic fluid

Initially, the variables were analyzed using bivariate logistic regression. Those with a *p*-value less than 0.05 were then included in a multivariate logistic regression to further explore their associations with the outcome variables. Among all the variables studied, the following were significantly associated with MSAF in multivariate analysis: gravidity = AOR: 3.643 (95% CI 1.215, 10.921), stage of labor, referring institution = AOR: 5.41 (95% CI, 2.053, 14.272), the time interval between decision and delivery = AOR: 1.361 (95% CI: 0.424, 4.365), obstetric complications = AOR: 6.820 (95% CI, 2.870, 16.202), eclampsia = AOR: 8.022 (95% CI, 1.634, 39.372), time taken from the referring institution to the managing institution = AOR: 15.258 (95% CI, 1.591, 146.328), cesarean section scar = AOR: 3.010 (95% CI, 1.344, 6.740), obstructed labor = AOR: 24.614 (95% CI: 6.073, 99.766), cephalopelvic disproportion = AOR: 2.640 (95% CI, 1.002, 6.950), fetal heartbeat abnormalities = [AOR: 2.068 (0.997, 4.292); AOR: 3.513 (95% CI, 1.633, 7.556)], and duration of labor = AOR: 5.44 (95% CI, 1.355, 9.782). Of these variables, only the stage of labor was found not to be associated with the outcome variables in the multivariate logistic regression (Table 4).

**Table 4:** Bivariate and multivariate analysis of factors.

Variables		Meconium stained amniotic fluid *N* (%)		COR (95% CI)	AOR (95% CI)	*p*-value
		No	Yes			
Gravidity	Gravida 1	141 (25.9)	198 (59.6)	3.225 (1.828,5. 5.692)**	**3.643** (**1.215,10.921)**	0.021
	Gravida 2–4	325 (59.9)	117 (35.2)	1.631 (0.926, 2.872)	1.805 (0.669,4.873)	0.244
	Gravida 5 and above	77 (14.2)	17 (5.2)	1	1	
Obstetric complications	Yes	127 (34.2)	31 (6.2)	1	1	
	No	244 (65.8)	473 (93.8)	2.113 (1.386,3.223)**	**6.820** (**2.870,16.202)**	0.00
Previous c/s scar	No	428 (71.3)	257 (93.5)	5.738 (3.447,9.551)**	**3.010** (**1.344,6.740)**	0.00
	Yes	172 (628.7)	18 (6.5)	1		
Stage of labor	Second stage	54 (9)	45 (16.4)	**2.627 (1.681,4.106)**	0.116 (0.033,.407)	0.23
	Active phase	174 (29)	112 (40.7)	2.029 (1.481,2.781)	0.945 (0.506,1.765)	0.45
	Latent phase	372 (62)	118 (42.9)	1	1	
Time interval b/n decision to delivery	>60 min	236 (39.3)	144 (58.8)	**2.789 (1.509,5.156)**	**1.361** (**0.424,4.365)**	0.05
	30–60 min	300 (50)	117 (47.8)	1.783 (0.962,3.303)	2.268 (0.670,7.678)	0.188
	<30 min	64 (10.67)	14 (5.1)	1	1	
Place of referral	Health center	44 (15)	40 (23.4)	2.006 (1.087,3.704)*	**5.41** (**2.053,14.272)**	0.001
	Government	185 (63.2)	102 (59.7)	**1.217 (0.737,2.008)**	1.587 (0.800,3.145)	0.186
	Private clinic	64 (21.8)	29 (16.9)	1	1	
Duration of labor	>24 h.	8 (1)	21 (7.6)	3.868 (2.313,6.470)*	**5.44** (**1.355,9.782)**	0.01
	<24 h	592 (99.99)	254 (92.4)	1	1	
Time taken from referring institution	>1 h	28 (46.8)1	169 (61.7)	1.827 (1.365,2.446)	**15.258** (1.591,146.328)	0.018
	<1 h	319 (53.2)	105 (38.3)	1	1	
Eclampsia	Yes	4 (0.7)	9 (3.3)	**5.041 (1.539,16.516)**	**8.022** (**1.634,39.372)**	0.01
	No	596 (99.3)	266 (9	1		
Obstructed labor	Yes	7 (1.2)	32 (11.6)	**11.156 (4.858,25.618)**	24.614 (6.073,99.766)^**^	0.00
	No	593 (98.8)	243 (78.4)	1	1	
Fetal heart beat	≤120	97 (16.2)	74 (26.9)	2.057 (1.439,2.941)^**^	2.068 (0.997,4.292)^**^	0.001
	≥160	112 (18.7)	56 (20.4)	1.348 (0.928,1.958)	**3.513** (1.633,7.556)	0.046
	120–160	391 (65.2)	145 (72.7)	1	1	
Cephalopelvic disproportion	Yes	12(2)	33(12)	**6.682 (3.394,13.155)**	**2.640** (**1.002,6.950)**	0.047
	No	588 (98)	242 (88)	1	1	

Bold values are those variables *p* value less than 0.05.

*p < 0.05.

**p < 0.01.

## 4 Discussion

The prevalence of meconium-stained amniotic fluid is 31.4%, which is relatively higher than in other studies in different parts of the world (10–12, 18, 22). The reason for this higher prevalence is that the study participants are mothers with emergency cesarean sections after the onset of labor, unlike the above studies, which included all mothers regardless of the route of delivery. This finding aligns with other scientific results from multiple studies, which showed that MSAF was strongly associated with an increased cesarean section rate (13, 16, 17, 22, 30), as well as being in labor, which is a strong risk factor for MSAF (31). Since the study participants are mothers for whom emergency cesarean sections were performed for different obstetric indications, a higher prevalence of MSAF is expected. Furthermore, this study showed that labor dystocia, such as cephalopelvic disproportion and obstructed labor, is strongly associated with the prevalence of MSAF. Pregnant mothers with CPD and obstructed labor have 2.6- and 24.6-times higher odds of having MSAF compared to mothers without dystocia. Technically, these labor abnormalities are related to a prolonged duration of labor, which is one of the factors associated with MSAF in this study.

The mothers with labor durations greater than 24 h have 5.44 times higher odds of developing MSAF than those with labor durations less than 24 h. Studies across the globe associate MSAF with labor abnormalities (dystocia) (11, 12, 14) as well as prolonged durations of labor (10, 11, 13, 31), which are the same as our findings. Hence, it has been proposed that the level of cortisol, higher during labor, is critically involved in stimulating bowel movement *in utero*, and the labor itself has an impact on stimulating bowel movement. Fetal cortisol levels rise in fetal plasma during labor, influencing the induction of intestinal motility. This was demonstrated in pregnant monkeys, where intra-amniotic injection of glucocorticoids led to the release of meconium (32). Since the aforementioned labor abnormalities are associated with prolonged labor, they predispose the fetus to stressful conditions that subsequently induce the release of meconium *in utero* (7, 8, 33).

Additionally, this study identified fetal heartbeat abnormalities, like tachycardia and bradycardia, as one of the determinants for MSAF. According to the findings, those mothers with fetal bradycardia and tachycardia are at a 2.0 and 3.5-fold risk of having MSAF when we compare them with those who have a normal fetal heartbeat. This finding is similar to various research (10, 12, 14–17). The proposed explanation for the release of meconium is that hypoxia *in utero* results in parasympathetic stimulation of bowel movement with subsequent release of meconium into the amniotic cavity (6, 29). Thus, fetal heartbeat abnormalities are a reflection of low fetal blood pH *in utero*.

According to this study, primigravida are 3.6 times highly likely to have an association with the prevalence of MSAF. This finding is supported by various studies which indicate that not having previous vaginal performances is a risk factor for MSAF (12, 17, 34). Based on detailed sociodemographic studies, however, the reason for the above finding is not clearly understood. But the likely mechanism is that primigravidity has an association with prolonged labor, especially the second stage than multigravidity; therefore, it has an indirect effect on MSAF (1, 35).

Obstetric complications and a history of previous cesarean sections are negatively associated with the study's outcome, except for eclampsia, which is positively associated with MSAF. In this study, pregnant women with obstetric complications, except for eclampsia, and a history of previous cesarean sections are 6.8 and 3 times less likely to have MSAF, respectively, than their counterparts. Among all obstetric complications, however, eclampsia had eight times higher odds of developing MSAF compared to those who did not. Despite the above results in this study, most literature associates obstetric complications as a predisposing factor for MSAF (10–12, 14, 15). The reason for this discrepancy may be that this study was conducted at tertiary hospitals where senior experts were directly involved in the management of obstetric complications, ensuring that pregnant women with obstetric complications were followed strictly and managed early with a low threshold for interventions.

The new finding in this study is the impact of institution-related factors like place of referral, the time taken to reach the managing health institution, and the time taken between the decision to delivery on the presence of MSAF. The pregnant women referred from the health center were more than five times more likely to have MSAF than those from the private clinic, where there was a strict follow-up and early referral. In the Ethiopian health structure, the health center is one of the lowest units, with a relatively low level of professionals and a lack of resources to diagnose as well as manage obstetric complications. On top of the above hurdles, health centers are commonly far away from referral hospitals, resulting in long-duration referral times. Accordingly, this study further strengthens the above facts by showing that pregnant women with a referral duration greater than 1 h are fifteen times more likely to have MSAF than those with less than one hour.

Moreover, the additional factor identified during this study is the interval between decision and delivery; as the interval increases to more than 60 min, the likelihood of developing MSAF becomes 1.36 times higher than that of less than 30 min. Globally, neonatal survival is increasing due to extensive work done in expanding advanced neonatal care during the past decades, although such improvement is not as expected in low-income countries (26). There is a lot to do in low-income countries through the expansion of advanced neonatal care service deliveries, infrastructure building, accessible roads, ambulance services, and resource supply, along with rigorous health professional training.

### 4.1 Limitation of the study

Because it is a cross-sectional study, it may not show the time association between the factors and the outcome variables. As this is the first study to use mothers who have undergone emergency cesarean sections as the study population, the authors based the sample size calculation on studies that focused on all routes of delivery.

### 4.2 Strength of the study

The authors tried to study additional factors like institution-related and intraoperative-related factors (though it has no association with the outcome variables) as well as the study population were who had emergency cesarean section.

## 5 Conclusion and recommendations

In this study, the prevalence of MSAF is relatively high. There are different factors associated with MSAF, including intrapartum-related factors like the duration of labor, obstructed labor, and cephalopelvic disproportion. Moreover, other factors include maternal obstetric complications, previous cesarean sections, and eclampsia. The last group consists of institution-related factors like the time to reach the managing institution, the place of referral, and the duration from decision to delivery. Therefore, an improvement in the quality of antenatal and intrapartum care is strongly recommended; professional development training and skill empowerment at health centers, which can provide comprehensive obstetric care, and strengthening the referral system are also suggested.

## Data Availability

The raw data supporting the conclusions of this article will be made available by the authors, without undue reservation.
